# Multiscale electronic and thermomechanical dynamics in ultrafast nanoscale laser structuring of bulk fused silica

**DOI:** 10.1038/s41598-020-71819-9

**Published:** 2020-09-16

**Authors:** Madhura Somayaji, Manoj K. Bhuyan, Florent Bourquard, Praveen K. Velpula, Ciro D’Amico, Jean-Philippe Colombier, Razvan Stoian

**Affiliations:** 1 Laboratoire Hubert Curien, UMR 5516 CNRS, Université de Lyon, Université Jean Monnet, 42000 Saint Etienne, France; 2 Academy of Scientific and Innovative Research, CSIR-Central Scientific Instruments Organization, Chandigarh, 160030 India; 3 Optical Devices and Systems Division, CSIR-Central Scientific Instruments Organization, Chandigarh, 160030 India

**Keywords:** Materials science, Nanoscience and technology, Optics and photonics, Physics

## Abstract

We describe the evolution of ultrafast-laser-excited bulk fused silica over the entire relaxation range in one-dimensional geometries fixed by non-diffractive beams. Irradiation drives local embedded modifications of the refractive index in the form of index increase in densified glass or in the form of nanoscale voids. A dual spectroscopic and imaging investigation procedure is proposed, coupling electronic excitation and thermodynamic relaxation. Specific sub-ps and ns plasma decay times are respectively correlated to these index-related electronic and thermomechanical transformations. For the void formation stages, based on time-resolved spectral imaging, we first observe a dense transient plasma phase that departs from the case of a rarefied gas, and we indicate achievable temperatures in the excited matter in the 4,000–5,500 K range, extending for tens of ns. High-resolution speckle-free microscopy is then used to image optical signatures associated to structural transformations until the evolution stops. Multiscale imaging indicates characteristic timescales for plasma decay, heat diffusion, and void cavitation, pointing out key mechanisms of material transformation on the nanoscale in a range of processing conditions. If glass densification is driven by sub-ps electronic decay, for nanoscale structuring we advocate the passage through a long-living dense ionized phase that decomposes on tens of ns, triggering cavitation.

## Introduction

Laser modification of bulk dielectric materials is the source of a significant range of applications, from the design of optical materials resistant to radiation to the fabrication of embedded optical devices and functions. Particularly refractive index engineering using ultrafast laser radiation is at the base of the development of three dimensional optical circuits capable of transporting and manipulating light^1,2^. To render the process efficient, the current challenge is to achieve index control in contrast and size with utmost precision. Recently, the utilization of non-diffractive irradiation concepts allowed to achieve characteristic processing sizes on the nanoscale^3,4^. The remarkable feature size below 100 nm for irradiation wavelengths in the near-infrared and the apparent bypass of the diffraction limit are related to a material response, notably a cavitation phenomenon^4^ in the presence of strong pressure gradients induced by the laser excitation. The existence of a material response enables thus to overcome the usual optical limits. Hence, material rupture scale defines the ultimate resolution, with sizes below the diffraction limit.

Relying on the extreme achievable scales, structures with high aspect ratio and nanoscale sections were used to fabricate hybrid micro and nanoscale features in bulk optical materials helping to design embedded systems with performant optical functions. Nanoscale features are for example useful to create read-out centers required to sample electrical fields in optical circuits or to generate strong optical resonances for Bragg sensors^5–7^, but equally they can serve as initiation centers for accurate cleavage of optical materials of technological importance^8–10^. Confined interactions were at the same time advocated for generating extreme conditions of pressure and temperature as the birthplace of novel material phases^11,12^. The achievement of high pressure levels and shock beyond the material mechanical resistance limit (tens of GPa in fused silica for example) is usually in competition with the rapidity of transformation into soft or liquid phases. It has been recently shown that in typical conditions of laser processing, void formation in fused silica occurs preferably by the early achievement of liquid phases and stretched liquid cavitation^13^. Fused silica is of prime interest in view of its technological importance and equally of its relevance as a model for fundamental light-matter interaction. Process dynamics in glasses with polarizable matrix in the presence of mobile charges (i.e. fused silica) was often discussed on ultrafast scales in terms of carrier rapid evolution and exciton trapping^14,15^, with less information on material long term evolution^16,17^. Electronic relaxation drives and, at the same time, is strongly dependent on the transient material evolution and particularly on its structural form, notably its passage via rigid or soft structural deformation phases.

We discuss here full-range electronic and thermomechanical evolutions of a fused silica material under volume confined irradiation using ultrashort laser Bessel beams in conditions of the achievement of either type I positive refractive index changes or, at the opposite, the formation of nanoscale voids. A first discussion on void formation was initially given in Ref.^13^ suggesting a slow, stress-driven cavitation phenomenon. This scenario will be confirmed and extrapolated here to a range of conditions generating nanoscale voids involving various focusing geometries and pulse durations, suggesting a common structuring mechanism in glass. Using time-resolved imaging of the interaction zones based on the employment of speckle-free low spatial coherence illumination, we visualize dynamically the heat flow and the material change over the whole relaxation cycle, down to the evolution arrest on microsecond scales with a cavitation process. We discuss the resulting conditions for structuring on scales much smaller than the optical wavelength. We equally monitor with ns time resolution the evolution of the plasma and extract temperature information of the interaction region, as well as electronic dynamics in strong excitation conditions. A comparison of plasma evolution in dense (silica) and rarefied (air) environments is illustrative for the process conditions and provides indication to the environment conditions for relaxation. This suggests electronic relaxation priming hydrodynamic evolution that becomes effective on tens of ns. Correlating spectral and imaging measurements, we provide a comprehensive view of electronic, thermodynamic and morphological material evolution for excited glassy matter in conditions of bulk confinement on multiple timescales, resulting on structuring at the nanoscale.

## Methods

Bulk fused silica samples (Corning 7980-5F, 800–1,000 ppm concentration of OH impurities) of parallelepipedic geometry were irradiated by near-infrared (800 nm) single laser pulses delivered from a regeneratively amplified ultrafast Ti:Sapphire laser system. The pulse duration is varied between 50 fs and 5 ps by adding additional second order dispersion via the compressor. A zero-order Bessel beam^18^ is generated using an axicon lens with an apex angle of 179°^19,20^. The resulting beam forms a narrow intense central core sustained over a long distance and surrounded by ring patterns, consequence of the conical interference of wavefronts of multiple orders induced by the conical phase at the axicon. The core is demagnified by a factor of 50 or 100 and imaged inside a *a*-SiO_2_ sample using a 4f afocal imaging system^3^ with a microscope objective as the final imaging element. Two types of focusing conditions determine the size of the Bessel beam inside the material. First moderate (NA = 0.20) focusing conditions were used (demagnification factor 50). The final core dimension is estimated at $\omega \simeq 1.5$ μm FWHM and the non-diffractive length stays in the $L=150$ μm range, ensuring a quasi one-dimensional character of the excitation geometry. Spatial filtering ensured no leakage from the axicon tip. The Bessel conical half-angle is $\theta =8^{\circ }$ in glass. Secondly, tighter focusing conditions were used, via a demagnification factor of 100 and an NA = 0.42 end optical element, resulting in a beam with $\theta =15^{\circ }$ and $\omega \simeq 0.8$ μm FWHM. Positive phase-contrast (PCM) microscopy is employed for observing the laser-generated structures in-situ. In PCM, phase shifts corresponding to negative and positive index changes appear bright and dark, respectively, on a grey background.

### Time-resolved microscopy

The complete relaxation dynamics is observed using time-resolved phase-contrast imaging. The observation covers the entire dynamic range until the evolution stop, following ultrashort pulse excitation of bulk fused silica in non-diffractive moderate and tight focusing conditions. We observe the laser-induced object in phase for a timescale ranging from ns to μs with sub-micron spatial resolution. A two-color pump-probe microscopy technique sensitive to relative differences in the object optical phase and having ns time resolution is used, collecting corresponding charts of transient material transformation at various illumination time moments down to the evolution stop. The pump pulse is represented by the 800 nm ultrashort laser pulse, shaped in the non-diffractive mode, creating the index change. The imaging setup is based on an upright diascopic optical microscope (Olympus BX-51) operating in optical transmission (OTM) and in phase-contrast mode (PCM). The observation objective NA = 0.55 gives a spatial resolution of 530 nm at the observation wavelength. For illumination, a stroboscopic method is used, and the probe pulse cross-illuminates the interaction zone. The illumination duration gives the temporal resolution of detection. To obtain high-resolution single-shot images with uniform background and low speckle noise, a low spatial coherence pulsed source is developed based on random lasing effect, as described in Ref.^21^, by creating a disordered gain medium. The laser gain medium consists of a colloidal solution of Rhodamine B (2.5 g/l) with immersed latex nanobeads (of 325 nm size) at a concentration of 4 × 10^15^ l^−1^. The random lasing effect is obtained by irradiating the colloidal solution with 532 nm laser pulses in the absorption band of the solution. Stimulated emission occurs where multiple scattering on the distributed nanobeads creates the random character. The exciting laser pulses in the solution are obtained from a frequency doubled Nd:YAG laser operating at 10 Hz and delivering 7 ns pulses in the mJ energy range. The random lasing effect is detected via the appearance of a strong spectral narrowing of the fluorescence band to around 13 nm bandwidth (FWHM) centered at 590 nm. Its pulse duration is similar to the excitation pulse in solution, i.e. 7 ns. A bright low-coherence illumination source has the potential of acquiring high quality speckle-free images^22^, providing at the same time information on the amplitude and on the phase of the object. This illumination method increases significantly the dynamical range of the image and the signal-to-noise ratio. The electronic synchronization between the ultrafast laser system and the ns laser system ensures the time-synchronization between the exciting ultrashort laser pulse in fused silica and the random lasing illumination (probe) source, with delays measured by a fast photodiode, permitting to correct for the jitter. The excitation region in the glass sample is imaged in a perpendicular geometry using a Köhler illumination arrangement. The probe enters the illumination path of the microscope via the microscope condenser and the optical transmission and phase-contrast microscopy images are recorded in the image plane with a back-illuminated electron multiplier Charge Coupled Device (EMCCD Andor iXon Ultra 897) camera. A 20 nm bandwidth pass filter centered at the probing laser wavelength (590 nm) is used to cut parasitic incoherent light emitted by the specimen and the scattering from the pump. Single pulses are selected with a shutter. Punctually, ultrafast sub-ps and ps dynamics data are used, recorded by the means of a standard pump-probe setup, where the probe is deducted from the pump, delayed with an optical delay line, and doubled in frequency before being inserted in the illumination path of the microscope^23^. The time-resolved microcopy technique covers a timescale ranging from sub-ps to μs.

### Gated plasma photoluminescence

For observing the luminescence from the excited region, an intensified gated Charge Coupled Device (Princeton Instruments ICCD PI-Max 3) was used to image and observe the interaction area directly coupled to the microscope port (imaging mode) or via a spectrometer (Acton) for spectral imaging. In this case the detector response time (gate width) determines the temporal resolution of the acquisition. A delay generator regulates the electronic time delay between the pump event and the acquisition moment. The zero-order of the spectrometer gratings permits the direct imaging of the interaction region, while the first diffraction order permit spectral analysis, all controllable via the motorized orientation of the grating. The internal optics of the microscope creates an artificial cutoff at around 390 nm. The spectral components around 800 nm were removed by a highly reflective mirror in this spectral band, with high transmission elsewhere in the visible range. The intermediate optical elements were spectrally calibrated using a deuterium-halogen light source. The gate, set to 5 ns, has a trapezoidal form. The delay time $\tau _D$ between the pulse and the acquisition is measured from the gate edge. As the yield is the integrated signal over the gate for each gate delay, this form has consequences on the representation of the detected signal. Depending on the signal length the signal maximum is recorded when the gate overlaps or overpasses the signal rise front. Due to the gate form artefact, for signals with a lifetime significantly larger than the gate, the maximum yield is shifted by up to 2 ns with respect to the case of a signal with a lifetime smaller than the gate.

Microscopy in phase-contrast mode and photoluminescence mapping, spectrally and spatially-resolved, will be dynamically associated to reconstruct the dynamics of laser interaction, the material states occurring during the process, and the development of the resulting index change.

## Results and discussion

### High refractive index structures

The first regime of interest relates to the onset of the positive (type I) refractive index changes. This interaction regime is primarily used for direct laser writing of optical waveguides in the bulk. It is usually achieved with very short (below 200 fs) low energy laser pulses, accumulating the effect after multipulse exposure. Generated in mostly moderate focusing conditions, this regimes relies on triggering intensity clamping which, via the emergence of light-scattering carriers, regulates and stabilizes the achievable peak intensity and carrier density. Typically, in these conditions, after ultrashort pulse laser excitation, a sub-critical plasma is formed which, in case of fused silica, relaxes rapidly (<150 fs) into self-trapped excitonic states (STE), with further evolution into defect states^23,24^. It has been deduced from spectroscopic studies that the bond-breaking processes will relax by promoting Frenkel pair defects [non-bridging oxygen hole centers (NBOHC) and holes localized on Si (E’ centers)] and by reforming Si-O annular structures of lower dimension^25,26^. The latter is equivalent to molecular agglomeration and densification. Thus compaction and defect formation are intrinsically related, originating from the same process of bond breaking and relinking. Time-resolved optical interferometry and imaging experiments have shown that carrier trapping is connected to the formation of a transient positive index change associated with the defect states^14,23,24^ as fast as 100 fs after excitation. The ultrafast pump-probe techniques are limited in the probing time-window to few ns and other slower processes may develop beyond this limit, thus monitoring processes down to the arrest of evolution is of interest. We first apply plasma diagnostics and microscopy to type I transformations in fused silica.

**Figure 1:**
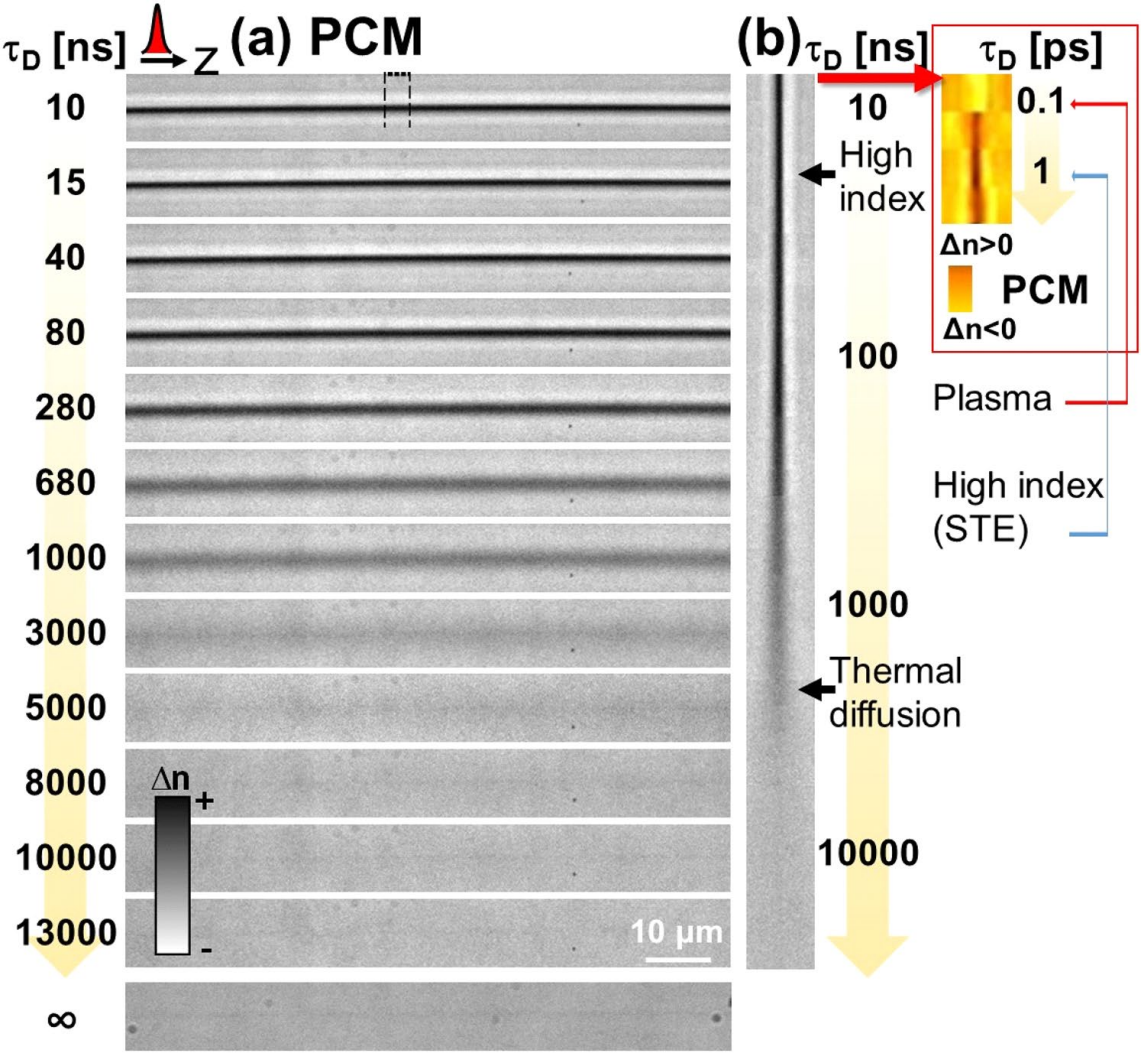
Evolution of laser-induced processes in type I positive index change conditions over the complete relaxation cycle. Moderate focusing conditions were used. The time-resolved phase-contrast microscopy setup was used with an electronically-synchronized pulsed illumination of 7 ns. (**a**) Time-resolved sequence of PCM images corresponding to the appearance of a permanent positive index change (dark colors) induced by a single Bessel laser pulse. The evolution is monitored for a time domain ranging from 1 ns to 13 μs. Input laser pulse parameters: 6 μJ and 50 fs. The laser beam arrives from the left. Scale bars are given for the relative index change. (**b**) Orthogonal views corresponding to the zone marked on the figure (**a**). For each time delay, regions of interest were selected and concatenated together to give a complete temporal perspective of each specific region. Transient modifications are indicated. A logarithmic timescale is used. The inset indicates ultrafast sub-ps processes as PCM data corresponding to the selected domain in (**a**), with fast decay of the carrier plasma into excitonic states. Data were extracted from Ref.^23^.

Investigating plasma relaxation, the gated ICCD plasma detection shows, expectedly, a negligible signal for single pulse type I conditions (with a weak detected yield in multipulse conditions that will be discussed later), consistent with the short carrier life time and small density of the electron population^23^. To observe the long term structural dynamics associated with this regime, the corresponding laser-excited object was monitored in phase and amplitude by the multiscale imaging technique based on microscopy. Given the weak amplitude signature of an index change, most of information comes from relative phase detection in PCM. Figure 1 shows the evolution of the excited region over a timescale of several μs. The type I structures are realized by single 50 fs laser pulses of 6 μJ energy in moderate focusing conditions. Figure 1a presents the raw sequence of images of the interaction region at different times. From this a stack of rectangular regions of interest (ROI) is selected (as represented in the figure) and concatenated together in an orthogonal view to render the dynamics more visible (Fig. 1b). Plotted on a logarithmic scale, this representation puts into evidence a transient high-contrast positive index change confined on the laser track. A diffusive character then appears, visualized as a transient enlargement, with a characteristic time of about 1 μs. This is the characteristic time of heat diffusion, consistent with a cooling time on the order of $w^2/D$, considering a thermal diffusivity of $D=0.9$ mm^2^/s and heat source transverse dimensions *w* in the range of 1 μm. The visualization of the heat flow is based on a strong thermo-optic effect in fused silica. The temperature variation of the refractive index reads as $dn=\partial n/\partial T\cdot dT$, with a positive thermo-optic coefficient (10^−5^ °C)^27^. After cooling, a permanent low-contrast narrow positive index trace remains. Here, photoluminescence and Raman spectroscopy studies indicated two characteristic features; a visible presence of NBOHCs accompanied by an increase of the number of three member rings in the structural glass matrix^26^. The results indicate that electronic modification processes at these low intensity regimes, typically considered as athermal, are nevertheless accompanied by heat. For completion, we recall here also the stages of ultrafast relaxation. The insert in Fig. 1b shows the ultrafast dynamics extracted from the data in Ref.^23^, plotted similarity on a logarithmic scale. It is thus interesting to note that a high index signature appears within 1 ps, almost simultaneous with the electron relaxation in about 200 fs. If the electronic decay originates from trapping into states self-induced by the electrons interaction with the matrix, the fast positive index onset (see discussion in Ref.^23^) was considered as emerging from an absorptive and dispersive signature of swift transient defect levels^24^ resulting from discrete energy levels in the bandgap or from structural distortions associated with these states. They are direct consequence of carrier trapping. The multiscale imaging suggests in parallel the involvement of a heat source that may as well form rapidly^28^, almost at the timescale of excitation in conditions of strong coupling between carriers and the silica matrix. It is to be noted that electronic trapping represents already a channel of energy transfer to the matrix and will accompany vibrational activation during collisional processes involved during absorption. These, as well as electronic screening, will affect the strength of electronic relaxation, and, with the increase of the dose, the relaxation develops slower components. From a certain level of excitation, a different interaction regime, more energetic and with stronger effects on matter, emerges.

### Nanoscale voids

If intensity clamping regimes (and the intrinsic intensity self-regulation) can be bypassed, concentrating more energy on the axis, a stronger interaction regime appears culminating with the generation of voids. In moderate focusing conditions, void formation is induced by stretching the pulse in time, that counterbalances the defocusing effect of the plasma. In the case discussed here an elongation to about 4 ps is used. Uniform void-like structures over the entire Bessel length can be obtained with cross-sections in the range of 100–400 nm^4^. Stretching the pulse in time will better confine energy bypassing the light-diffusing character of the carrier plasmas^4,23^ while keeping a sufficiently high nonlinear excitation cross-section. The energy concentration can increase ten fold with respect to the short pulse exposure. The achieved volume energy density and the resulting gradients from energy confinement overcome the material strength and determine the appearance of the void in a one-dimensional geometry. This energy concentration has consequences on plasma dynamics and on the material morphological evolution. We note that a similar situation appears in conditions of tight focusing, using only ultrashort laser pulses; higher energy pulses generate type II changes without the requirement to stretch the pulse, as focusing in this case is strong enough to counteract the effect of diffraction and plasma defocusing.

#### Plasma dynamics

**Figure 2:**
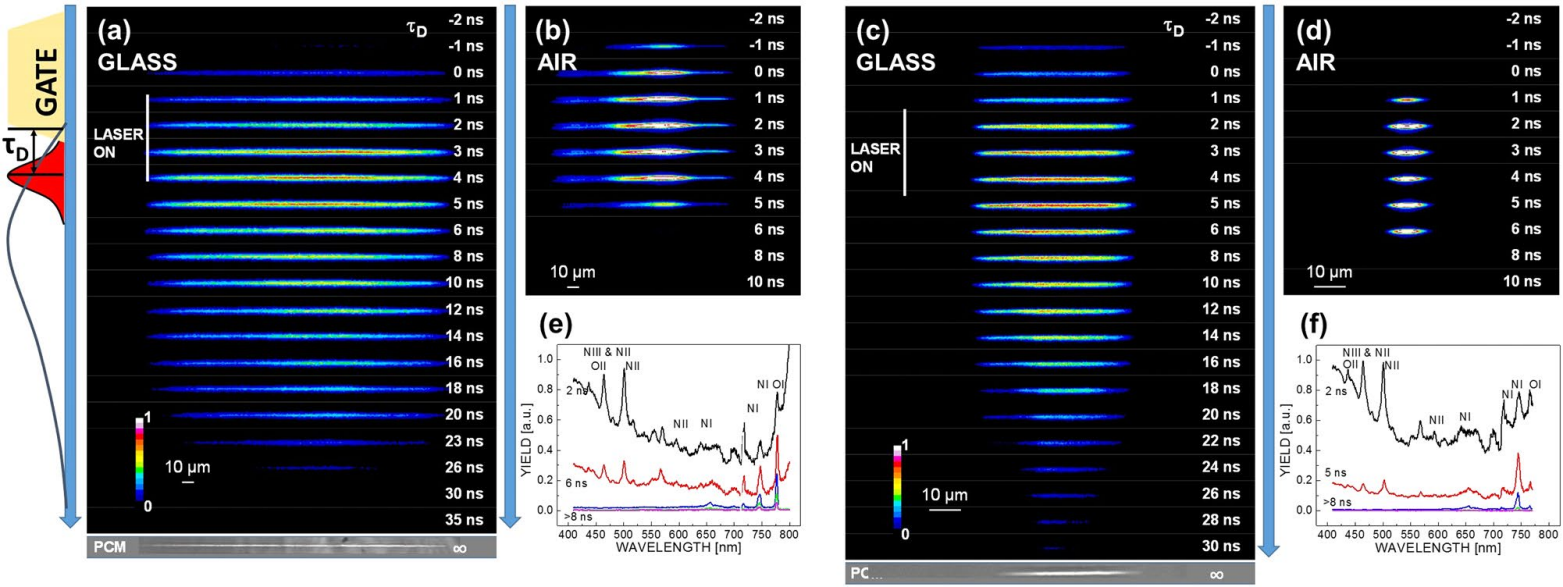
The evolution of the plasma in the interaction region in conditions corresponding to void formation. Time-resolved spectrally integrated images are given. (**a**) Photoluminescence imaging in fused silica in moderate focusing conditions. (**b**) Photoluminescence imaging in air in moderate focusing conditions. Irradiation parameters: fused silica: 10 μJ, 3 ps and air: 20 μJ, 3 ps. (**c**) Photoluminescence imaging in fused silica in tight focusing conditions. (**d**) Photoluminescence imaging in air in tight focusing conditions. Irradiation parameters: fused silica: 7 μJ, 100 fs, and air: 20 μJ, 100 fs. The acquisition is made in an imaging geometry using a gated ICCD with a 5 ns gate. $\tau _D$ represents the interval between the gate edge at half-maximum and the laser pulse. Thus the emission maxima of glass and air are offset by 2 ns. The zone where the gate fully overlaps with the laser pulse is marked as “laser on”. The relative time shift between the emission maxima in air and silica is due to the trapezoidal form of the gate (see text for details). (**e**) Photoluminescence spectrum in air and its time evolution (moderate focusing conditions). (**f**) Photoluminescence spectrum in air and its time evolution (tight focusing conditions).

The nature of the excited glass state can be approached by observing the associated plasma. This gives not only an estimation of the strength of energy deposition but provides elements defining the way the energy is being transferred to the glass matrix. To gain insights into the characteristics of the excited phase, we have performed plasma luminescence imaging and spectral imaging for Bessel excitation in silica glass, and comparatively, in air. The reason is to observe plasma evolution in dense and rarefied environments. A gated ICCD camera was used with a gate window of 5 ns. The first measurement is made in a direct imaging mode, collecting the spectrally integrated but spatially resolved luminescence. Figure 2 shows the temporal evolution of the laser-induced plasmas in conditions leading to void generation in both moderate and tight focusing regimes. Their yield is significant, approximately thousand times more than the residuals observed in multishot type I conditions, suggesting a stronger excitation range. However, the most important observation concerns the luminescence lifetime. The glass volume excitation in moderate conditions with stretched and energetic pulses (3 ps, 10 μJ per pulse) shown in Fig. 2a) results in a luminescence evolution with a lifetime exceeding 10 ns (with a peak, given the definition of $\tau _D$, at 4 ns). This luminescence results most probably from Bremsstrahlung radiation emitted by hot carriers. This is no longer consistent with an ultrafast self-trapping mechanisms and a different, much slower recombination mechanism is at work. A second hypothesis relates the signal to the thermoluminescence of the hot glass. These hypotheses will be discussed in view of current scenarios of shock and rapid rarefaction^11^ that foresee a rapid cavitation and density decrease in less than 1 ns. To check the assumption of a rarefied excited gas, we performed comparative studies of photoluminescence in air (moderate focusing conditions, 20 μJ, 3 ps). Figure 2b shows a luminescence signal of excited air comparable to the gate, suggesting a lifetime smaller than the gate duration. Being comparable to the gate, the short time range of the signal suggests that air plasma luminescence is only present when sustained by the fs laser radiation, with a rapid decay following up. A respectively similar behavior was measured in tight focusing conditions in glass (7 μJ per pulse, 100 fs) and air (20 μJ per pulse, 100 fs), confirming the different evolutions in condensed and rarefied phases. These are represented in Fig. 2c,d. To identify the air emitting species, a time-resolved spectral imaging procedure was applied. The spectrum was recorded via the microscope using a spectrometer and the gated ICCD with variable time delay. The corresponding air breakdown spectra are given in Fig. 2e,f, for conditions similar to Fig. 2b,d. The cutoff around 400 nm is generated by the intermediary imaging optics of the microscope. The spectra confirm the rapid decay of photoluminescence, with a signal evolution comparable to the gate width and some low intensity residuals. Contrary to reported air filament spectra showing fluorescence of nitrogen molecular lines in conditions of intensity clamping^29^, these breakdown spectra consist dominantly of ionized nitrogen lines, NII and NIII, and oxygen OII lines, as well as atomic NI and OI lines. The high yield is a laser-supported emission, where ionization is sustained by the laser radiation. A simulation of the spectrum based on Saha equation^30^ mapping the ratio NIII to NII emission yields using as reference the 463.36  NII line, the 464.45 nm NIII line, the 500.4 nm NII line, and the 567.9 nm NII line suggests peak electronic temperatures in the range of $T_e=3-4$ eV and densities of $N_e=10^{16}-10^{18}$ cm^−3^. This seems confirmed by the apparent presence of OII line at 434 nm. The gate integration time covers the whole relaxation stage and the appearance of NI and OI lines (746.8 nm and 777.3 nm respectively) in the red part of the spectra, with their reminiscence at late times, are signaling a cooling phase with still high electronic densities and temperatures in the range of $T_e=1$ eV.

**Figure 3:**
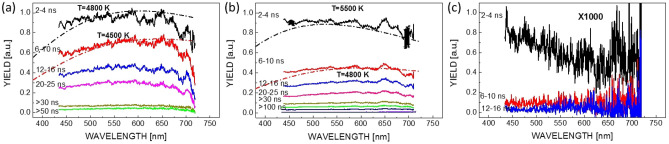
The evolution of the glass plasma in the interaction region in conditions corresponding to void formation and index increase. (**a**) Void conditions for moderate focusing geometry. Time-resolved plasma spectra are given at different time moments, together with fitting Planck distributions. (**b**) Void conditions for tight focusing geometry. Time-resolved plasma spectra are given at different time moments, together with fitting Planck distributions. Error bars are included. The yield was calibrated accounting for the response of the optical elements and the quantum efficiency of the detector. Irradiation conditions are similar to Fig. 2. (**c**) For comparison multishot type I spectra (note that single shot type I conditions did not allow sufficient spectral yield to be recorded).

Spectral detection was equally applied for the excited bulk silica in both moderate and tight focusing conditions. For signals with long lifetime, the results represent the spectral yield integrated over the gate time, much longer than the excitation pulse. The spectra are continuous, without discrete lines, and the spectral dynamics is shown in Fig. 3. Moderate and tight focusing conditions generating embedded voids are given in Fig. 3a,b, where the maximum yield appears at a delay time of 4 ns in our measuring configuration. The time evolution is equivalent to that already described in Fig. 2a,c. In spite of detection uncertainties (for example modulations due to partially uncompensated optical response of the 800 nm rejection filter), the spectra show a quasi-flat and continuous spectral signature with a slight peaking in the visible range. This pertains mostly the peak spectra when the laser pulse is still on (the gated detection records excitation phases sustained by the laser pulse), feeding energy absorption and generating thus non-equilibrium. A curved convex shape appears at latter times. In the hypothesis that the observed photoluminescence comes from Bremsstrahlung radiation from hot carriers following collisional regimes of deceleration in the plasma phase, the concentration of carriers seems sufficiently high to begin transforming flat Bremsstrahlung typical shapes due to self-absorption into black-body distributions^31^. Thus the spectra can be fitted with a Planck distribution $I_{\lambda }=\varepsilon _{eff} 8 \pi c h \lambda ^{-5} (e^{h c/k T \lambda }-1)^{-1}$, with $\varepsilon _{eff}$ being the effective emissivity, *T* the temperature, and the rest, the usual quantities. Best fits were obtained for temperatures between 4,500 and 5,500 K for the ns scale dynamics observed in this case during and just after the peak, the range being below the critical value. Within this frame, the electronic population seems quasi-equilibrated with the matrix, creating an excited condensed phase. The temperature values provide indication that nucleating a gas-phase is not particularly efficient^32^. With time the signal goes slowly down in intensity without a significant shift towards larger wavelengths, indicating that during the decay phase there is no significant and swift rarefaction, nor strong cooling. The latter observation may be the consequence of self-absorption, preserving partly the energy of the plasma. A similar result was obtained in Ref.^33^ for bulk irradiation with energetic Gaussian pulses. On surfaces irradiated with ns laser pulses, significantly higher temperatures were reported^34^. Comparing the air and bulk glass results we can conclude that, in both scenarios, carrier Bremsstrahlung or thermoluminescence, we deal in the glass case with a yet not rarefied solid that experiences a hot, excited, and dense phase for several tens of ns. The density and carrier concentrations appear to be sufficiently high (albeit the lack of quantitative values) to reinforce self-absorption and to follow a dynamic behavior different from that of a rarefied excited gas, which cools down immediately after exposure.

The character of the excited state is equally different with respect to the soft type I excitation. For comparison, type I spectra acquired in multishot conditions are equally given (Fig. 3c), where the signal is recorded for a given delay time over several shots on the same spot in conditions generating soft positive index changes. As noted before, single shot type I conditions did not allow sufficient spectral yield to be recorded. Due to the level of the signal and in view of the uncertainties of the measurement, we only focus on the level of the signal and not on the spectral shape. This indicates a residual plasma being formed; no particular photoluminescence bands from defects can be recognized in view of the low yield. Concerning to the general plasma behavior, it is of interest to know if this behavior is general or it is limited to the particular test case of fused silica. This will be a topic for further research. The specific relaxation via a hot excited dense fluid phase has thermomechanical consequences resulting in the final type of modification.

**Figure 4:**
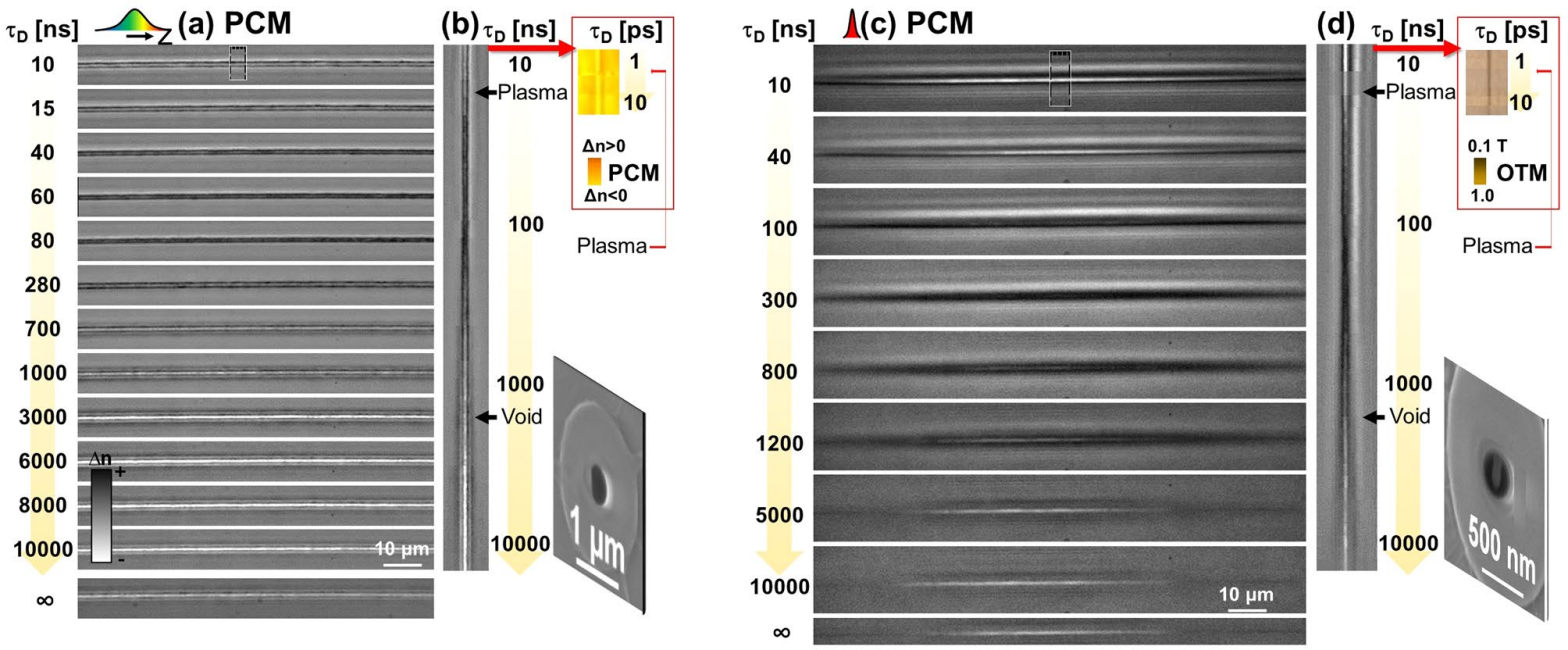
Evolution of laser-induced processes in type II void-generation conditions over the entire relaxation cycle. (**a**) Multiscale time-resolved sequence of PCM images corresponding to the appearance of a void-like structure (light colors) induced by a single laser pulse. The evolution is monitored for a time domain ranging from 1 ns to 10 μs, with imaging delays given on the side. Input laser pulse parameters: 17 μJ and 5 ps. The laser pulse comes from the left. Scale bars are given for the relative index change, indicating the modification sign. (**b**) Orthogonal views corresponding to the zone marked on the figure. For each time delay, regions of interest were selected and concatenated together to give a complete temporal perspective of each specific region. Transient modifications are indicated. A logarithmic timescale is used. The inset indicates early stages of plasma formation extracted from the PCM data in Ref.^23^ corresponding to the selected domain in (**a**). (**c**,**d**) Multiscale dynamics similar to (**a**,**b**) but in tight focusing conditions. (**c**) Time-resolved sequence of PCM images corresponding to the appearance of a void-like structure (light colors) induced by a single laser pulse. (**d**) Orthogonal views corresponding to the zone marked on the figure. The different domains were cropped and concatenated to give a time perspective on a logarithmic scale. Input laser pulse parameters: 2 μJ and 50 fs. The inset indicates early stages of plasma formation extracted from the OTM ultrafast dynamics data for the selected region in (**c**). The measurement is similar to the data reported in Ref.^13^.

#### Multiscale phase imaging of thermomechanical evolution

The characteristic plasma decay has consequences on the material transformation dynamics. To access the thermomechanical evolution in fused silica in conditions of void formation, we have followed the material evolution over the whole material relaxation range using the time-resolved phase contrast microscopy. The technique delivers the evolution in space and time of the relative refractive index change. The succession of the processes is given in Fig. 4 as phase objects. Figure 4a shows the evolution of the optical phase signal of the Bessel-modified glass trace. Irradiation conditions are: 17 μJ pulse energy and 5 ps pulse duration for moderate focusing. As before, a rectangular ROI is selected for each delay time and the resulting stacks are concatenated together offering an orthogonal projection in a logarithmic timescale (Fig. 4b). The full-cycle monitoring technique reveals a complex sequence of processes. At the beginning a low-index phase with a lifetime of about 100 ns is observed, recognizable by the white color phase shift. This can be attributed to the presence of free carriers. The inset present the ultrafast imaging range, confirming the steady presence of the plasma^23^. This is consistent with the results shown above on plasma luminescence and confirms the electronic origin of the luminescence. Then, the excited material relaxes into a higher index phase. The transient high contrast positive index change is then characteristic of a hot phase. The subsequent slow opening of the void (white colors) suggests that, in this material and processing frame, a liquid form is formed, undergoing cavitation^13^. Resulting from cavitation, the permanent modification size lies well below the diffraction limit (see inset). The same behavior is obtained in tight focusing conditions using ultrashort laser pulses^13^, and the observations discussed in Ref.^13^ are used to illustrate, reinforce, and extrapolate the nanostructuring scenario over the whole range of conditions. The subsequent dynamics is depicted in Fig. 4c,d as a succession of PCM images (irradiation conditions 2 μJ and 50 fs) and the inset gives optical transmission microscopy ultrafast data. The figure shows equally the nanoscale transverse dimension of the obtained embedded void.

The slow dynamics is intriguing and we recall that under energetic irradiation conditions faster (sub-ns) shock-driven solid-phase micro-explosion scenarios were proposed^11,12^ with much faster evolution to rarefied domains. Thermo-elasto-plastic calculations for the solid state^35^ support this dynamics as a result of sudden pressure increase. In the present case we speculate that the specific behavior observed here, namely the slow development of the void, is intrinsically related to the dynamic competition between mechanical relaxation and phase-like transformation, i.e. the achievement of the liquid phase, associated with a potentially rapid sub-ps heating mechanism (witnessed by the lack of trapping). Along the rapid achievement of the liquid phase, the Young modulus decreases, relaxing the pressure constraints in the bulk before explosion, with the liquid cavitating at later times, driven by stress at the interface on the cooling phase. A similar behavior was observed in tight focusing conditions^13^. The involvement of a liquid column was confirmed by a transition from uniform voids to fragmented domains driven by hydrodynamic instabilities in soft low viscosity phases^13^. This indicates on one hand the importance of a material response in achieving structuring sizes significantly smaller than the wavelength and the capability of beam engineering in triggering and controlling such response and the associated relaxation dynamics.

## Conclusion

In conclusion we have observed the electronic and thermomechanical relaxation dynamics over the entire evolution cycle for laser modifications corresponding to positive index changes and to void formation in silica glass. A dual perspective based on imaging and spectroscopy confirms nanostructuring scenarios and extrapolates the process over a large range of conditions. A fused silica test material was chosen in view of its technological importance as well as the in view of the relevant physics related to strongly-coupled glassy systems. A multitude of physical processes were dynamically followed, from the initial plasma phase down to thermal and mechanical dissipation of energy. We have shown that previously reported electronic processes in type I index change conditions, usually associated with transient electronic states, are accompanied by a certain amount of heat. The STE and heat signatures overlap in time and suggest a potentially rapid heating mechanism due to electron-matrix coupling. We equally interrogated strong interaction regimes resulting in material rupture and generation of voids, where both dispersion engineering and the focusing strength contribute to energy confinement. In laser processing conditions, void formation relates in fused silica to a slow liquid cavitation process. This ensures equally the nanoscale dimension of the void. The initiating plasma phase shows long lifetime in the tens of ns range, with temperatures of the excited region around 5000 K. This is consistent with a rapidly-formed liquid phase, not yet decomposing mechanically, that is further ionized. Comparison with excited gases is given to endorse the scenario resulting from the competition between pressure relaxation and heating with softening. Indicative information, complementing dynamic imaging, results from different plasma dynamics in dense and rarefied environments, supporting the scenario of a silica relaxation within a dense phase. These observations are important to understand volume laser processing on scales much smaller than the processing wavelength.

## References

[1] Gattas RR, Mazur E (2008). Femtosecond laser micromachining in transparent materials. Nat. Photonics.

[2] Sugioka K, Cheng Y (2014). Ultrafast lasers–reliable tools for advanced materials processing. Light: Sci. Appl..

[3] Bhuyan MK (2010). High aspect ratio nanochannel machining using single shot femtosecond Bessel beams. Appl. Phys. Lett..

[4] Bhuyan MK (2014). Single-shot high aspect ratio bulk nanostructuring of fused silica using chirp-controlled ultrafast laser Bessel beams. Appl. Phys. Lett..

[5] Martin G (2017). Near infrared spectro-interferometer using femtosecond laser written GLS embedded waveguides and nano-scatterers. Opt. Express.

[6] Zhang, G., Cheng, G., D’Amico, C. & Stoian, R. Efficient point-by-point Bragg gratings fabricated in embedded laser-written silica waveguides using ultrafast Bessel beams. *Opt. Lett.* **43**, 2161–2164 (2018). 10.1364/OL.43.002161 29714779

[7] Ertorer E, Haque M, Li J, Herman PR (2018). Femtosecond laser filaments for rapid and flexible writing of fiber Bragg grating. Opt. Express.

[8] Mishchik K (2017). Improved laser glass cutting by spatio-temporal control of energy deposition using bursts of femtosecond pulses. Opt. Express.

[9] Rapp L (2017). High speed cleaving of crystals with ultrafast Bessel beams. Opt. Express.

[10] Jenne M (2018). Glass cutting optimization with pump-probe microscopy and Bessel beam profiles. Proc. SPIE.

[11] Juodkazis S (2006). Laser-induced microexplosion confined in the bulk of a sapphire crystal: evidence of multimegabar pressures Phys. Rev. Lett..

[12] Vailionis A (2011). Evidence of superdense aluminium synthesized by ultrafast microexplosion. Nat. Commun..

[13] Bhuyan, M. K. *et al.* Ultrafast laser nanostructuring in bulk silica, a “slow” microexplosion. *Optica* **4**, 951–958 (2017).

[14] Papazoglou D, Tzortzakis S (2008). In-line holography for the characterization of ultrafast laser filamentation in transparent media. Appl. Phys. Lett..

[15] Bergner K (2018). Spatio-temporal analysis of glass volume processing using ultrashort laser pulses. Appl. Opt..

[16] Sakakura M, Terazima M (2004). Oscillation of the refractive index at the focal region of a femtosecond laser pulse inside a glass. Opt. Lett..

[17] Hayasaki Y, Fukuda S, Hasegawa S, Juodkazis S (2017). Two-color pump-probe interferometry of ultra-fast lightmatter interaction. Sci. Rep..

[18] Durnin J, Miceli JJ, Eberly JH (1987). Diffraction-free beams. Phys. Rev. Lett..

[19] Mcleod J (1954). The axicon: a new type of optical element. J. Opt. Soc. Am..

[20] Indebetouw G (1989). Nondiffracting optical fields: some remarks on their analysis and synthesis. J. Opt. Soc. Am. A.

[21] Mermillod-Blondin A, Mentzel H, Rosenfeld A (2013). Time-resolved microscopy with random lasers. Opt. Lett..

[22] Hokr HB (2017). Enabling time resolved microscopy with random Raman lasing. Sci. Rep..

[23] Velpula PK (2016). Spatio-temporal dynamics in nondiffractive Bessel ultrafast laser nanoscale volume structuring. Laser Photon. Rev..

[24] Martin P (1997). Subpicosecond study of carrier trapping dynamics in wide-band-gap crystals. Phys. Rev. B.

[25] Chan J, Huser T, Risbud S, Krol D (2003). Modification of the fused silica glass network associated with waveguide fabrication using femtosecond laser pulses. Appl. Phys. A.

[26] Mishchik K (2013). Modification of the fused silica glass network associated with waveguide fabrication using femtosecond laser pulses. J. Appl. Phys..

[27] Ghosh G (1995). Model for the thermo-optic coefficients of some standard optical glasses. J. Non-Cryst. Solids.

[28] Jürgen P, Vrakking MJJ, Stoian R, Husakov A, Mermillod-Blondin A (2019). Plasma formation and relaxation dynamics in fused silica driven by femtosecond shortwavelength infrared laser pulses. Appl. Phys. Lett..

[29] Xu HL, Azarm A, Bernhardt J, Kamali Y, Chin SL (2009). The mechanism of nitrogen fluorescence inside a femtosecond laser filament in air. Chem. Phys..

[30] https://www.nist.gov/pml/atomic-spectra-database.

[31] Ghisellini, G. Radiative processes in high energy astrophysics. *Spinger: Lecture Notes in Physics*, 873 (Springer, Heidelberg, 2013).

[32] Rudenko A, Colombier JP, Itina TE (2018). Nanopore-mediated ultrashort laser-induced formation and erasure of volume nanogratings in glass. Phys. Chem. Chem. Phys..

[33] Qian J (2016). Ultrashort pulsed laser induced heatingnanoscale measurement of the internal temperature of dielectrics using black-body radiation. Appl. Opt..

[34] Carr CW, Radousky HB, Rubenchik AM, Feit MD, Demos SG (2004). Localized dynamics during laser-induced damage in optical materials. Phys. Rev. Lett..

[35] Beuton R (2018). Thermo-elasto-plastic simulations of femtosecond laser-induced multiple-cavity in fused silica. Appl. Phys. A.

